# Long-term survival in metastatic gastric cancer patient with Apatinib plus S-1 maintenance treatment following first-line chemotherapy—case report

**DOI:** 10.3389/fonc.2024.1478719

**Published:** 2024-11-18

**Authors:** Zhen Wang, Hao Li, Yue Guan, Mingshuang Wu, Manqing Hu, Chenchen Fang, Huaxing Wu, Maopeng Yang

**Affiliations:** ^1^ Endoscope Department, Harbin Medical University Cancer Hospital, Harbin, Heilongjiang, China; ^2^ Department of Medical Oncology, Harbin Medical University Cancer Hospital, Harbin, Heilongjiang, China; ^3^ Department of Medical Oncology, Fourth Affiliated Hospital of Harbin Medical University, Harbin, Heilongjiang, China; ^4^ Department of Medical Oncology, Xiaogan Hospital Affiliated to Wuhan University of Science and Technology, Wuhan, Hubei, China

**Keywords:** gastric cancer, Apatinib, S-1, oxaliplatin, maintenance treatment

## Abstract

**Aim:**

Gastric cancer is the third leading cause of cancer-related mortality and the fifth most common cancer globally. In China, many patients are diagnosed at an advanced stage and cannot be operated on, so the prognosis is very poor. The role of maintenance therapy after first-line chemotherapy in gastric cancer is still unclear. Apatinib is a small-molecule VEGFR-2 TKI and is currently approved for third-line treatment after failure of second-line chemotherapy for advanced gastric cancer. In this article, we use case reports to illustrate its effectiveness and to study what effective maintenance treatments are available for gastric cancer.

**Methods:**

The main treatment is chemotherapy and targeted therapy. We reported a 68-year-old man with a diagnosis of advanced gastric cancer with liver metastasis. From 12/2014 to 05/2015 Oxaliplatin + S-1(dose) chemotherapy for 6 cycles, the efficacy of partial response (PR) evaluation. After the patient’s oral S-1 + Apatinib (dose) to maintain the therapeutic usage and dosage, the patient is well tolerated, the tumor is reviewed regularly, and the condition is stable. The patient was finally infected with the COVID-2019 in May 2023, resulting in death after ineffective treatment.

**Results:**

After Oxaliplatin combined with S-1 regimen, the effect was up to PR. S-1 combined with Apatinib maintenance therapy after first-line chemotherapy, the patient has survived for more than 7 years without progression.

**Conclusions:**

The current treatment of advanced gastric cancer is not satisfactory. The combined application of S-1 and Apatinib in gastric cancer maintenance therapy deserves further study. Maintenance therapy for gastric cancer has attracted wide attention, and more large-scale clinical research is under way.

## Introduction

Gastric cancer is the fifth most common malignancy in the world and ranks as the fourth leading cause of cancer-related death. Half of the world’s gastric cancer patients are in East Asia (1). The main treatment for gastric cancer is surgery, but many patients have lost their chance of surgery at the time of treatment. For metastatic gastric cancer, chemotherapy and targeted therapy are the main treatments (2), but the prognosis is very poor, with an average survival of less than 1 year (3). Maintenance therapy after first-line chemotherapy for advanced tumors has benefited patients with advanced colorectal cancer and non-small cell lung cancer, and the value of maintenance therapy in gastric cancer has not been determined. Apatinib is a recently developed small-molecule TKI targeting VEGFR-2, a third-line treatment for advanced, metastatic gastric cancer in China for second-line chemotherapy failure. In addition, we want to report that a patient with advanced metastatic gastric cancer undergoes first-line S-1 and Oxaliplatin chemotherapy, and after partial tumor remission, S-1 combined with Apatinib maintenance therapy, and progression-free survival has been more than 7 years, and the quality of life is well.

## Case presentations

**Figure 1:**
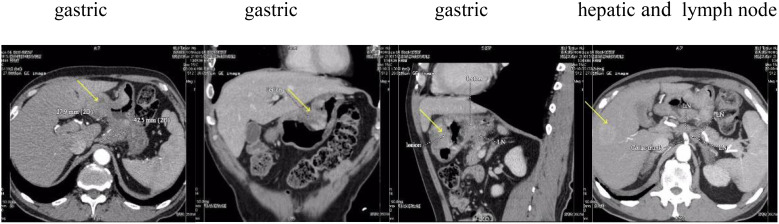
December 2014, examination without treatment. Yellow arrows indicate the location of the lesion.

**Figure 2:**
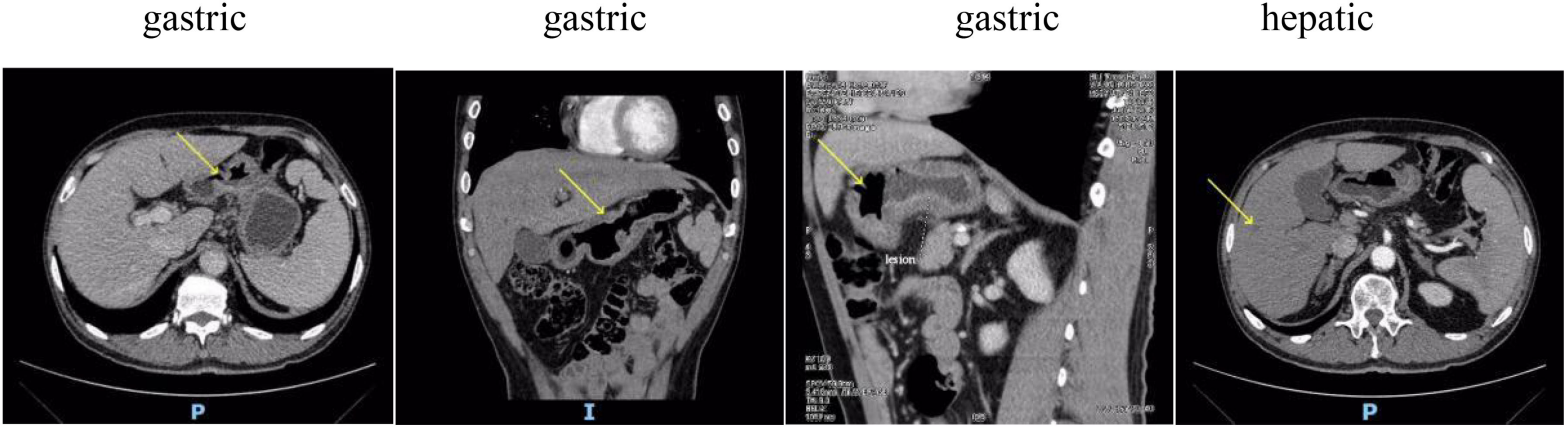
June 2015, CT after six cycles of Oxaliplatin combined with S-1 chemotherapy. Yellow arrows indicate the location of the lesion.

**Figure 3:**
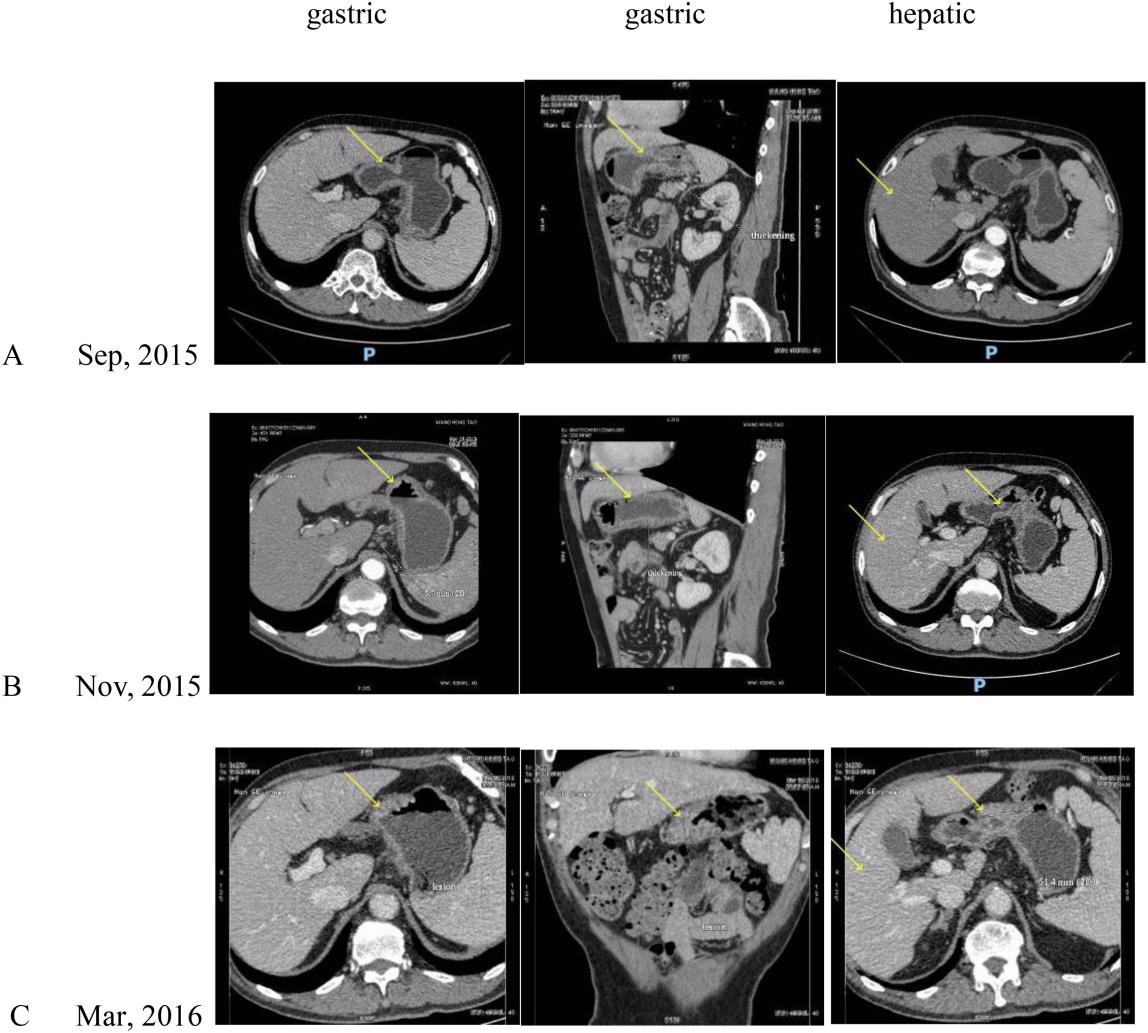
**(A)**: The stomach and liver were scanned by CT at the first follow-up after S-1 monotherapy. **(B)**: The stomach and liver were scanned by CT at the second follow-up after S-1 monotherapy. **(C)**: CT of lesion progression. Yellow arrows indicate the location of the lesion.

**Figure 4:**
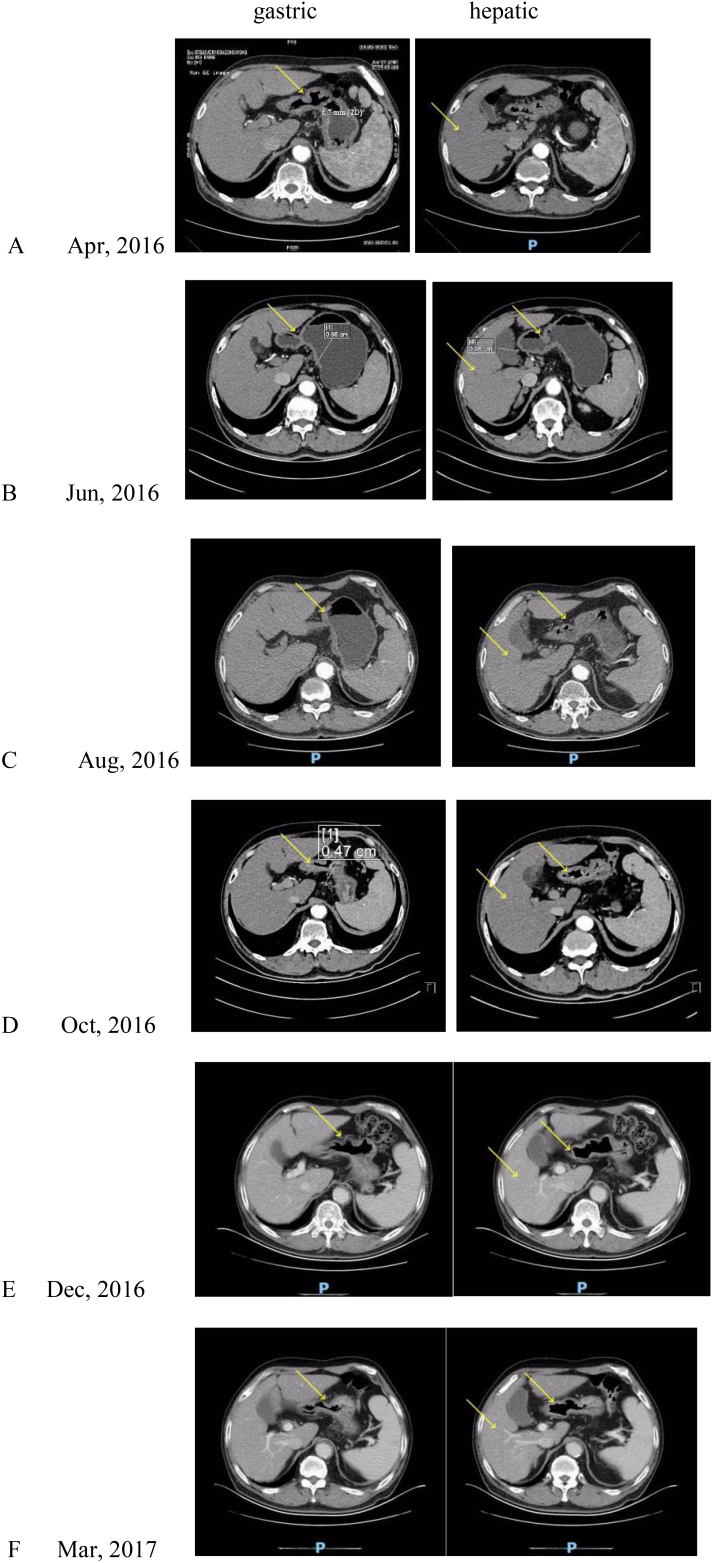
**(A)**: CT after 1 cycle of Apatinib, **(B)**: CT after 2 cycles of Apatinib with S-1 chemotherapy, **(C)**: CT after 4 cycles of Apatinib with S-1 chemotherapy, **(D)**: CT after 6 cycles of Apatinib with S-1 chemotherapy, **(E)**: CT after 8 cycles of Apatinib with S-1 chemotherapy, **(F)**: CT after 11 cycles of Apatinib with S-1 chemotherapy. Yellow arrows indicate the location of the lesion.

**Figure 5:**
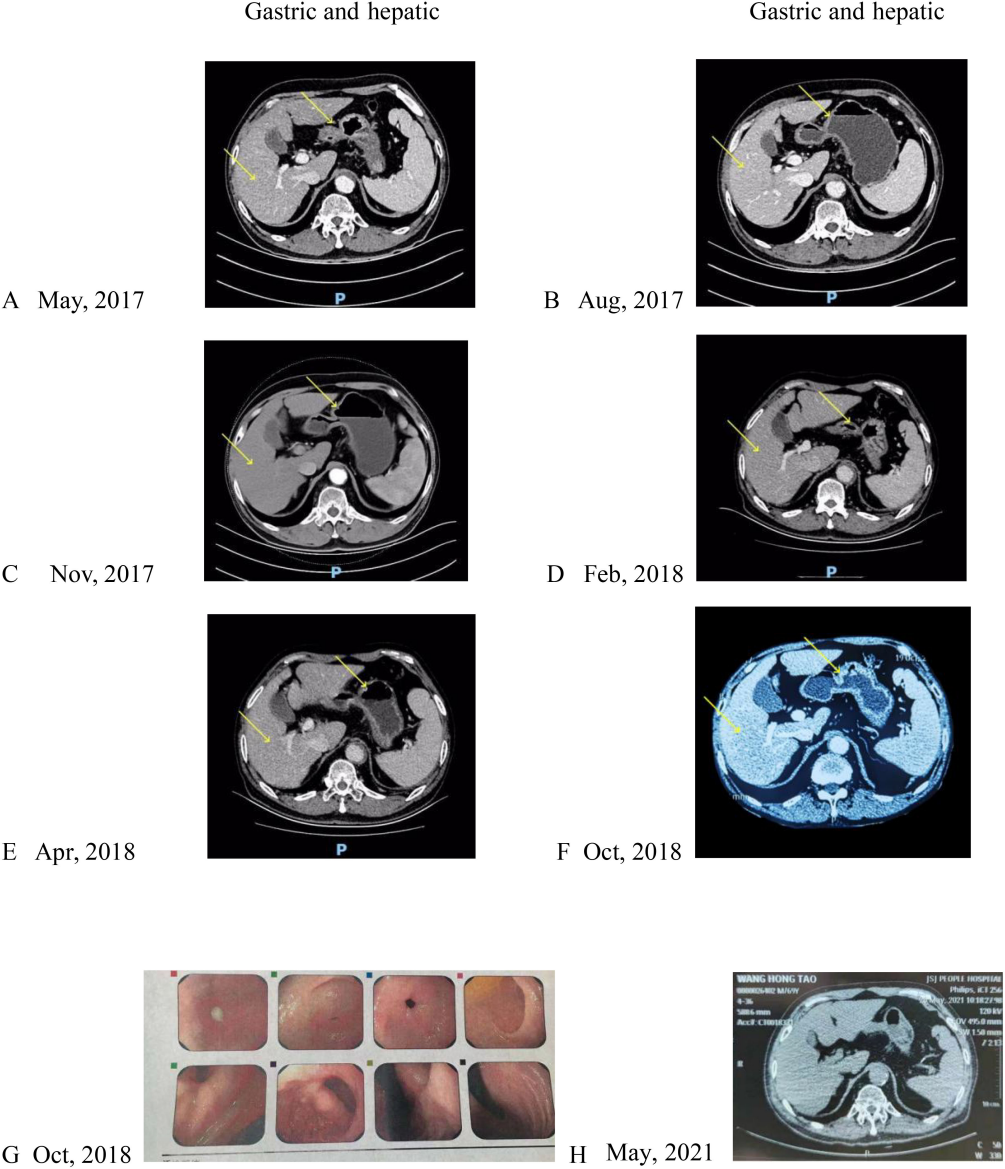
**(A)**: CT after 2 cycles of maintenance therapy with Apatinib monotherapy, **(B)**: CT after 5 cycles of maintenance therapy with Apatinib monotherapy, **(C)**: CT after 8 cycles of maintenance therapy with Apatinib monotherapy, **(D)**: CT after 11 cycles of maintenance therapy with Apatinib monotherapy, **(E)**: CT after 13 cycles of maintenance therapy with Apatinib monotherapy, **(F)**: CT after 19 cycles of maintenance therapy with Apatinib monotherapy, **(G)**: gastroscopic results showed that the gastric lesions were CR, **(H)**: CT examination after drug withdrawal. Yellow arrows indicate the location of the lesion.

A 68-year-old male patient with bloating, vomiting, and loss of appetite. In December 2014, the gastroscope was seen in the lower part of the stomach, the stomach angle, and most of the antrum of the stomach was multiple ulcers, and the mucosal edema around the ulcer was congested. Take pathology: poorly differentiated adenocarcinoma. Immunohistochemistry showed the following: human epidermal growth factor receptor 2 (HER-2) negative. Stomach 3D + full abdominal CT (**Figure 1**, December 2014) showed gastric cancer Borrmann type 3, hepatic multiple metastatic tumors, and multiple lymph node metastasis in the abdominal cavity and retroperitoneum. The tumor marker is normal. After six cycles of systemic chemotherapy with Oxaliplatin+S-1 (tegafur/gimeracil/oteracil potassium) from 12/2014 to 06/2015, the patient reviewed a full abdominal + stomach 3D CT (**Figure 2**, June 2015), which showed the PR of stomach lesion and hepatic metastatic lesion. Although the patient achieved PR during treatment, after multiple cycles of high-dose treatment, the patient was in poor physical condition with insufficient scores. Considering the patient’s humanistic care and quality of life, as well as the need to live with dignity, the patient altered to have oral S-1 monotherapy for maintenance therapy from 06/2015 to 03/2016 (**Figure 3**). The patient was admitted on 15/03/2016 for review, and the full abdominal + stomach 3D CT showed PD in gastric lesions and a hepatic metastatic lesion. The patient was in a state of cachexia due to poor physical condition and could not have second-line treatment of gastric cancer. The patient had an alternative of Apatinib treatment, oral dose 250/500 mg, with alternate oral therapy every other day. The stomach 3D + full abdominal CT (**Figure 4**, April 2016) showed the lesions in the stomach and liver were significantly reduced, and the treatment effect was PR. The treatment effect of metastatic lymph nodes around the stomach was PR. After the review in 04/2016, due to the markedly improved physical condition, the patient received Apatinib treatment, oral dose 250/500 mg, with alternate oral therapy every other day combined with S1 plan. S1, 40 mg/20 mg, alternate every day, 2 times a day. The stomach 3D + full abdominal CT (**Figure 4**, June 2016) indicated that reduced stomach lesion, and the medical imaging results achieved CR, and the lesions in the liver were reduced, and the treatment effect was PR. The treatment effect of metastatic lymph nodes around the stomach was PR. The stomach 3D + full abdominal CT (**Figure 4**, August 2016) indicated a markedly reduced stomach lesion, and the medical imaging results achieved CR. No obvious lesions were observed in enhanced CT of hepatic metastatic lesion, and the treatment effect of hepatic metastatic lesion was CR. The treatment effect of metastatic lymph nodes around the stomach was PR. The stomach 3D + full abdominal CT (**Figure 4**, October 2016, December 2016, March 2017) indicated that the medical imaging results of stomach lesion CR. No obvious lesions were observed in enhanced CT of hepatic metastatic lesion, and the treatment effect was CR. The treatment effect of metastatic lymph nodes around the stomach reached PR. Patients were given maintenance therapy with Apatinib monotherapy, oral dose 250/500 mg, with alternate oral therapy every other day. The stomach 3D + full abdominal CT (**Figure 5**, May 2017–April 2018) indicated that the medical imaging results of stomach lesion CR. No obvious lesions were observed in enhanced CT of hepatic metastatic lesion, and the treatment effect was CR. The treatment effect of metastatic lymph nodes around the stomach reached PR. On 24 October 2018 (**Figure 5**), the whole abdominal CT and the gastroscopic results of the patient indicated that the treatment effect of gastric lesions was CR, enhanced CT of liver metastases showed no obvious lesions, and the treatment effect of perigastric metastatic lymph nodes reached CR. So the patient stopped taking the drug. The patient developed lower extremity artery occlusion on 15 January 2020 and was treated with a stent. On 20 May 2021 (**Figure 5**), the abdominal CT showed that the treatment effect of gastric lesions reached CR, enhanced CT showed no obvious lesions in liver metastases, the treatment effect reached CR, and the treatment effect of perigastric metastatic lymph nodes reached CR. The patient was in good condition, and the overall survival was 106 months. Patients with the final in May 2023 new coronavirus infection, cause of death after active treatment is invalid.

## Discussion

It is generally believed that palliative chemotherapy for advanced gastric cancer can prolong survival and improve quality of life compared with BSC, but even so, the prognosis of metastatic gastric cancer is still poor, with an average OS of about 1 year (3). The standard first-line treatment currently recommended for advanced gastric cancer is a two-drug regimen of fluorouracil and platinum for some patients who are younger, have better physical conditions, or have a chance of transformation (4). A three-drug program can also be considered. This case is an elderly male; according to the guidelines and the patient’s condition, the oxaliplatin combined with the S-1 regimen is selected. At the end of the sixth cycle of chemotherapy, the patient has a good therapeutic effect, the tumor is obviously relieved, and the adverse reaction is mild. The optimal duration of first-line chemotherapy for advanced gastric cancer is unknown. The treatment strategies used in different phase III studies are inconsistent until the disease progresses, after a predetermined period of withdrawal or reduced maintenance therapy, but in clinical practice, tolerability often precludes continuing combination chemotherapy until progression (5, 6).

The fundamental purpose of maintenance therapy is to maintain a small adverse response while prolonging the patient’s PFS and OS. The efficacy of maintenance therapy after first-line chemotherapy for advanced tumors has been recognized in non-small cell lung cancer (7) and colorectal cancer (8). G. Stocker et al. Studied platinum-fluoropyrimidine-based induction therapy and first-line chemotherapy for HER-2 negative gastric cancer, patients with stable disease were treated with S-1. The results showed an OS of 16. 6 months (80% CI, 13. 0–17. 2 months) and a median PFS of 7. 4 months (80% CI, 7. 1–9. 1 months). The authors believe that S-1 follow-up maintenance therapy can bring survival benefits (9).

Gong et al. First-line treatment of advanced gastric cancer with paclitaxel Plus Capecitabine (PX), Capecitabine monotherapy was continued for patients without disease progression. PFS and OS in all patients were 188 and 354 days, respectively. Forty-five patients were treated with Capecitabine monotherapy. The patient’s OS was significantly prolonged (531 days). Adverse reactions to maintenance therapy were tolerated, hand-foot syndrome was the main toxicity that restricted the use of Capecitabine (10). Park SR et al. compared the efficacy of continuous SOX until progression (continuous arm) or to have a chemotherapy-free interval followed by SOX reintroduction at progression (stop-and-go arm), after the first-line chemotherapy of the six-cycle S-1/Oxaliplatin (SOX) regimen in patients with metastatic gastric cancer. The results showed that progression-free survival (PFS) was significantly longer in the continuous arm than in the stop-and-go arm (10. 5 vs. 7.2 months; hazard ratio [HR] 0.55, 95% CI, 0.37–0.81; *P* = 0.002). Median duration of disease control (DDC), 10.5 versus 11.3 months, HR 0.92 (95% CI, 0.62–1.36; *P* = 0.674); median OS, 22.6 versus 22.7 months, HR 0.78 (95% CI, 0.50–1.23; *P* = 0.284). Adverse events including grade ≥3 fatigue (28.8% vs. 8.1%; *P* = 0.003) and sensory neuropathy (25.4% vs. 9.7%; *P* = 0.022), The author of the article believes that maintenance treatment can improve PFS but cannot improve DDC and OS (11). Qiu et al. to study Capecitabine as maintenance treatment after first-line chemotherapy using Oxaliplatin and Capecitabine in advanced gastric adenocarcinoma patients, the median progression-free survival (PFS) was 11.4 months for maintenance treatment patients, while 7.1 months for the control group, *P* < 0.001. The multivariate analysis showed that the maintenance treatment was an independent prognostic factor in advanced gastric adenocarcinoma patients (12). LU B et al. conducted a prospective study of Capecitabine as maintenance therapy after Capecitabine-based combination chemotherapy for patients with advanced esophagogastric junction adenocarcinoma. The results showed that the OS/PFS of the Capecitabine maintenance treatment group was significantly longer than that of the control group (13).

In conclusion, the optimal duration of first-line chemotherapy for advanced gastric cancer is still unknown. It is still unclear whether patients with better first-line treatment should be treated with maintenance therapy and which regimen is the optimal. Limited clinical evidence suggests that maintenance therapy with oral fluorouracil appears to benefit patients, but the results are not consistent, and existing clinical studies have small sample sizes and poor quality. Large-scale, high-quality randomized controlled trials are needed to address this problem. At present, the problem of maintenance treatment of gastric cancer has caused widespread concern, and more large-scale clinical research is underway.

S-1 is consisting of tegafur, 5-chloro-2, 4-dihydroxypyridine (CDHP), and potassium oxonate (Oxo) at a 1:0. 4:1 molar ratio. CDHP was used as a potent reversible inhibitor of 5-Fu degradation. Oxo can reduce the gastrointestinal toxicity of 5-Fu (14). Meta-analysis showed that S-1 can provide survival benefit compared with 5-Fu-containing regimens for advanced gastric cancer (15).

A phase II study showed that in patients with advanced gastric cancer, S-1 had an ORR of 44% and an OS of 207 days, with high safety and no adverse events of grade 4 or higher (16). Further studies have shown that S-1 combined with platinum drugs can achieve higher efficacy than S-1 monotherapy, so this has become one of the standard protocols for chemotherapy for advanced gastric cancer in China and Japan (17, 18). In addition, the S-1 combined cisplatin with Herceptin regimen can also be used in the HER-2 positive advanced gastric cancer with good efficacy and safety, and the median overall survival was 14.4 months. The 1- and 3-year overall survival rates were 66.7% (19).

The development of targeted therapy for gastric cancer is still unsatisfactory. Currently, only a few targeted drugs such as Herceptin have been approved for the treatment of advanced gastric cancer, which may be related to the heterogeneity of gastric cancer. Angiogenic drugs can overcome the problem of tumor heterogeneity to some extent because they act on new blood vessels, to date ramucirumab (20, 21) and Apatinib (22) have been approved for advanced gastric cancer treatment.

Apatinib is a novel oral small molecule VEGFR-2 pathway inhibitor that selectively inhibits VEGFR-2 tyrosine kinase activity, inhibits the proliferation of vascular endothelial cells that depend on this pathway, and reduces microvascular density in tumors to inhibit tumors growing (23), and Apatinib can inhibit tyrosine kinase activity in the RET/Src pathway (24). In addition to standalone inhibiting tumor growth, *in vivo* studies have found that Apatinib can circumventing multidrug resistance (MDR) and improve the therapeutic efficacy of chemotherapy (25, 26).

In a phase III trial (27), 267 patients with advanced gastric or gastric-esophageal junction adenocarcinoma were enrolled, for whom two or more prior lines of chemotherapy had failed. Patients were randomly assigned to oral Apatinib or placebo. Median OS was significantly improved (6.5 months vs. 4.7 months, *P* = 0.0149; HR, 0.709; 95% CI, 0.537 to 0.937; *P* = 0.0156). Apatinib significantly prolonged median PFS (2.6 months vs. 1.8 months; *P* < 0.001; HR, 0.444; 95% CI, 0.331 to 0.595; *P* < 0.001). The most common grades 3–4 nonhematologic adverse events were hand-foot syndrome, proteinuria, and hypertension. Subsequent studies have shown that in advanced gastric cancer in China, 500 mg/day of Apatinib can achieve almost the same efficacy as 850 mg/day, and the toxicity is significantly reduced (28).

Clinical study of Apatinib combined with S-1 in the treatment of patients with advanced gastric cancer. The ORR, DCR, median OS and median PFS of Apatinib + S-1 group were 9.5% (4 of 42), 71.4% (30 of 42), 257 days, and 123 days. Respectively, the ORR, DCR, median OS, and median PFS in the S-1 group were 0% and 40.5% (17 of 42), 234 days and 67 days. The efficacy of Apatinib + S-1 group was significantly higher than that of S-1 group. However, the incidence of hypertension, proteinuria, and hand-foot syndrome was significantly higher in the simple group (28).

In this study, after Oxaliplatin combined with S-1, the effect was up to PR. S-1 combined with Apatinib maintenance therapy after first-line chemotherapy, the patient has survived for more than 7 years without progression. Such a remarkable PFS was achieved, in the first place due to the sensitivity of patients to chemotherapy drugs and Apatinib treatment. The second is that we found that in the study of Scott, there was no significant difference in the mPFS of oral Apatinib 250 or 500 mg/day (22). Tian et al. showed that the lower dose of oral Apatinib was associated with fewer adverse reactions, better patient tolerance, a higher disease control rate, and better quality of life in patients with advanced gastric cancer (29), and considering the patient’s physical state, we took Apatinib 250/500 mg and alternate oral secondary treatment plans every other day. The second-line treatment and low dose therapy reduced the patient’s adverse reactions and extended the patient’s survival period. At the same time, we began to study the role of VEGFR, MVD and other indicators in the prognosis of Apatinib and explore the reasons for the 7-year PFS achieved by Apatinib maintenance therapy.

In conclusion, the current treatment of advanced gastric cancer is not satisfactory. In addition to the development of new drugs, the exploration of new treatment models should also pay attention. Maintenance therapy has observed survival benefit in patients with lung and colorectal cancer. The drugs for maintenance therapy should be selected with the curative effect, the adverse events are tolerable, the cost is low, and it is convenient to use. The combination of S-1 and Apatinib meets this requirement, and its role in the maintenance treatment of gastric cancer deserves further study.

## Data Availability

The original contributions presented in the study are included in the article/supplementary material. Further inquiries can be directed to the corresponding authors.
